# Learning to exploit a hidden predictor in skill acquisition: Tight linkage to conscious awareness

**DOI:** 10.1371/journal.pone.0179386

**Published:** 2017-06-20

**Authors:** Randy Tran, Harold Pashler

**Affiliations:** Department of Psychology, University of California, San Diego, San Diego, California, United States of America; Western University, CANADA

## Abstract

It is often assumed that implicit learning of skills based on predictive relationships proceeds independently of awareness. To test this idea, four groups of subjects played a game in which a fast-moving “demon” made a brief appearance at the bottom of the computer screen, then disappeared behind a V-shaped occluder, and finally re-appeared briefly on either the upper-left or upper-right quadrant of the screen. Points were scored by clicking on the demon during the final reappearance phase. Demons differed in several visible characteristics including color, horn height and eye size. For some subjects, horn height perfectly predicted which side the demon would reappear on. For subjects not told the rule, the subset who demonstrated at the end of the experiment that they had spontaneously discovered the rule showed strong evidence of exploiting it by anticipating the demon's arrival and laying in wait for it. Those who could not verbalize the rule performed no better than a control group for whom the demons moved unpredictably. The implications of this tight linkage between conscious awareness and implicit skill learning are discussed.

## Introduction

A critically important aspect of skill acquisition is learning to take advantage of the various predictive relationships that exist within the relevant domain. Through reinforcement learning and other learning processes, people are assumed to discover and exploit such predictive relationships and to optimize their performance accordingly, achieving greater rewards as their skill grows (e.g., [1]). The question posed in the present article is: do people learn to exploit predictive relationships without showing any conscious awareness of the relationship that they are exploiting?

Anyone acquainted with the cognitive psychological literature might suppose that the answer to this question is clearly “yes”. Indeed, there are several well-known lines of research which seem to show beyond any doubt that implicit procedural learning takes place without conscious awareness (see [2], for a review; c.f., [3]). Moreover, in the past 15 years or so, rather little in the way of new research on the topic seems to have been published, possibly suggesting that the issue has seen by many as “settled”. However, as will be seen below, these results, though intriguing, involve very limited kinds of behavioral changes that are not necessarily representative of skill learning in the broader sense. The remainder of this introduction provides a brief overview of research demonstrating unconscious procedural learning, pointing out how these studies leave open the general question posed above. We then go on to describe the construction of a very simple videogame designed expressly to revisit and shed light on the question posed here.

### Evidence for unconscious implicit learning

A number of experimental designs have produced results that appear to show extensive implicit learning without awareness. In the best known of these studies, Willingham, Nissen, and Bullemer [4] had people make a series of button pushes in response to a spatial sequence of stimuli, and repeated a 10-item sequence throughout the experiment. Subjects were divided into 3 different groups depending on their ability to explicitly describe the repetition. Most subjects showed a speed-up in performance for repeated sequences, but were unable to verbally describe the sequences that repeated. While the reduction in RT with practice was greater for subjects who could describe the repeated sequence, there was substantial improvement even for those who could not describe it (for discussion, see [5–9].)

Another commonly cited line of evidence for learning of predictive relationships without awareness comes from Miller [10,11], who had subjects respond to the identity of a central “target” characters while ignoring some other “flanker” characters that were presented on either side of the central character. The identity of the flanker characters partially predicted the identity of the target character. Subjects responded faster on trials that conformed to this predictive relationship, as compared to trials that deviated from it ([10,11]). Several pieces of evidence argued for a dissociation between awareness of the predictive relationship and behavioral reliance upon the relationship. For example, a small proportion of subjects were unable to report the most common flanker-character pairing, and for these “unaware” subjects the flanker effect on response latencies was actually *stronger*, rather than weaker, than for other subjects (although the difference was not statistically significant.)

While these results would appear to suggest that unconscious implicit learning is probably ubiquitous, the research designs represent only a rather narrow set of behavioral changes compared to the typical real-world skill acquisition challenge that people face. For one thing, the choice reaction-time tasks used in some studies required people to respond quickly and accurately to the stimuli that were only partially predictable based on the covert relationship (in the Willingham et al. [8] design, that was based on the identity of the previous stimulus; in the Miller [10,11] studies, that was the identity of the flanker.) Exploiting the presence of these stimuli may therefore have involved tuning the perceptual system itself to lower the threshold for identifying stimuli likely given the context. This seems quite different than choosing overt actions taken based on anticipations of predictable future events. It is also possible that people might have failed the tests of awareness given by these investigators because these tests required them to produce information that was valid only on some proportion of trials (subjects might not comment on a regularity they had consciously noticed at one point in learning if they had later observed apparent disconfirmation of the regularity).

Moreover, the repetition of the stimulus series in the Willingham, Nissen, and Bullemer [8] design also meant that a sequence of responses was repeated. Subjects may have formed higher level "chunks" of motor programs that represented multiple finger responses. These may be poorly verbalized precisely because they represent motor response patterns (just as we would not expect people to be well able to verbalize what they do when they play ping-pong.)

### Empirical challenges to unconscious learning

Another set of studies from Lewicki and colleagues would appear to be more directly on point for the question posed above—and relatively immune to the objections raised in the preceding paragraph. Lewicki and colleagues published numerous studies that purported evidence for learning hidden covariations (e.g., [12]; see also [13] for a review.). For example, in Hill et al. [12], subjects were shown faces that covaried facial features and personality characteristics (e.g., “fair professors always had ‘long’ faces…unfair professors always had ‘short faces’.”) in the training phase. Next, subjects rated the “fairness” of novel faces in the testing phase. The data showed that subjects tended to rate novel long faces to be fair and novel short faces to be unfair (Hill et al. [12], Experiment 1). However, the subjects were unable to verbalize the hidden covariation in their exit survey and were therefore said to have acquired it unconsciously. The evidence from Hill et al. suggest unconscious acquisition of hidden covariation can be exploited for future events.

Unfortunately, however, there is reason to doubt the replicability of these studies. Hendrickx, de Houwer, Baeyens, Elen, and Van Avermaet [14] attempted 9 conceptual and 3 direct replications and only one of these efforts confirmed the original finding. Hendrickx et al. [14] suggested that their replication attempts were actually better controlled than the original studies (i.e., minimized correlated features) and had more sensitive awareness measures (e.g., a recognition questionnaire with elaboration instead of free response). Hendrickx et al.’s [14] replication of the described study showed that only subjects who were able to describe the hidden covariation in the exit survey showed the predicted pattern of results (e.g., long faces rated as fair, short faces rated as unfair). Subjects who were unable to describe the covariation did not show this effect. Given the large number of non-replications, we would suggest that the results of Lewicki and colleagues should not be assumed valid. Moreover, the Hill et al. [12] designs also provided only very coarse measures of awareness; in the studies described here, we provide a more fine grained temporal window on this process.

### Current approach

As described above, most past studies of implicit learning of predictive regularities have focused on very austere choice-reaction time designs in which subjects' task is merely to make one of a small number of button-press responses chosen in compliance with a fixed stimulus-response mapping provided by the experimenter, or on tasks in which people make repeated sequences of motor responses. We felt that by moving to a computer game environment we could examine the effect of embedding hidden predictive relationships on a more robust and compelling example of skill learning.

The very simple videogame used in both experiments described below was designed with two specific aims in mind. One aim was to introduce a predictive regularity that would be valid on 100% of trials, to reduce the likelihood that people would abandon whatever conscious access they might achieve due to encountering contrary cases. Second, rather than relying on subtle latency changes due to priming as in the studies described above, the game was devised so that detection of a regularity would allow the participant to improve his or her score by making active behavioral choices in anticipation of what would happen next in the game environment.

## Experiment 1

**Fig 1:**
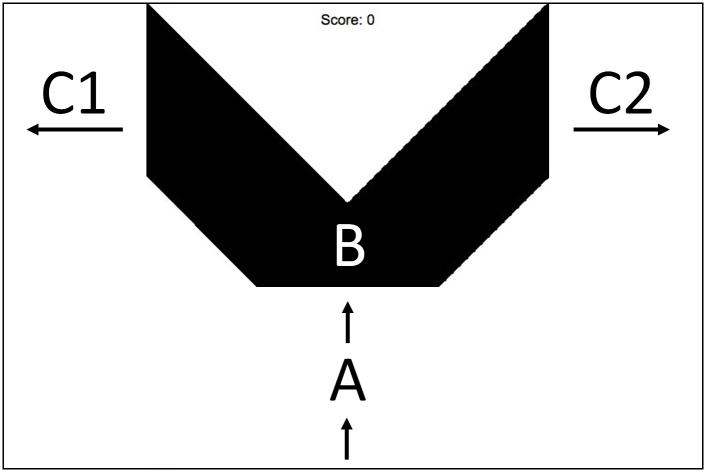
Example of the game screen. General appearance of the screen viewed by subjects in the “Demon Hunting Game”. The “demon” is paused at the bottom of the screen (A), waiting for the subject to click on it. When the subject does this, the demon resumes its upward trajectory which takes it behind the black V-shaped occluder (B), whereupon it finally emerges moving more rapidly in the location labeled C1 or C2. At this point, the subject must click on the demon before it leaves the screen in order to score. Neither the lines nor the letters are present on the game screen. Unbeknownst to some groups of subjects, the height of the demon's horns predicts whether the demon reappears on the left or right side.

The simple computer game used in Experiment 1 worked as follows (Fig 1). Each play began with a simple cartoonlike figure (a “demon”) moving upward from the bottom of the screen to the location shown in Fig 1 (point A). At this point, the demon came to a rest, and remained there until the subject placed the computer mouse cursor over the demon and clicked on it. The purpose of this requirement was to insure that subjects fixated briefly on the demon. Once the mouse click was registered, the demon resumed its upward motion, moving behind a large black V-shaped occluder (point B in Fig 1). After 3 seconds, it re-emerged, either on the left side of the screen heading leftward (point C1 in Fig 1), or on the right side of the screen heading rightward (point C1 in Fig 1). In this phase, it was now moving quite fast (217 pixels/sec), and the player's challenge was to click on the demon before it left the screen. If they could do so, they would earn one point. Subjects performed up to 180 separate “plays”, each lasting about 10.5 seconds. Between blocks of 30 plays, subjects were required to rest for 20 seconds without opening any other browser windows (their scores on all blocks of play completed thus far were displayed during this rest period.)

Pilot work in our lab looking in detail at behaviors emerging in this game showed that subjects who were told the rule found it easy to reach near-perfect scoring levels on every trial, and that they accomplished this (as expected) by moving the cursor in advance to the place where the demon was to reappear, and laying in wait for it. Unlike the graded latency changes seen in the implicit learning literature described above, this strategy did not produce a mere subtle modulation of response latency, but rather a change in strategy resulting in a drastic improvement in the rate of scoring (raising performance from not much better than 50% up to nearly 100% in most cases).

In Experiment 1, subjects were randomly assigned to one of four groups. For the first group (Control), whether the demon went left or right was random and not predictable based on any property of the demon. Thus for the Control Group, reliably anticipating the location of reappearance was impossible. For Groups 2–4, the height of the demon's horns perfectly predicted where the demon would reappear: long horns meant that it would go left and short horns meant that it would go right. The difference in horn height was a very salient 5:1. For Group 2 (Predictable/No Instruction Group), the instructions did not mention anything about the predictive relationship. The final two groups of subjects were told either simply that horn height would be relevant (Dimension Instruction Group) or they were given a precise description of the exact rule (Full Instruction Group).

The experiment was divided into 6 blocks of 30 plays. All subjects were told that if they were able to score a point on *every single play* within a given block of 30, their participation would be complete at the end of the block, and they would be paid as soon as they answered a few final questions (“exit interview”). In this exit interview, subjects in all groups (except the Full Instruction group) who reached the performance threshold were asked if they had any hunches enabling them to predict which way the demon would go. The opportunity to be excused from the study after a perfect-scoring block served two purposes: it motivated the subjects to do as well as they could, and it insured that exit interviews took place only a short time after the moment at which the subject first demonstrated mastery of the game.

## Method

### Participants

Subjects were drawn from our laboratory’s on-line research subject pool, which provides a diverse panel of subjects of various ages from a wide variety of countries. The subjects provided written informed consent, and the research was approved by the University of California San Diego Social and Behavioral Sciences Institutional Review Board. Subjects are pre-screened for comprehension of English, careful attention to instructions and conscientious performance in prior experiments. A total of 97 subjects completed the study in return for payment of $6.00. Subjects were randomly assigned to one of the four between-subject conditions.

### Stimuli

**Fig 2:**
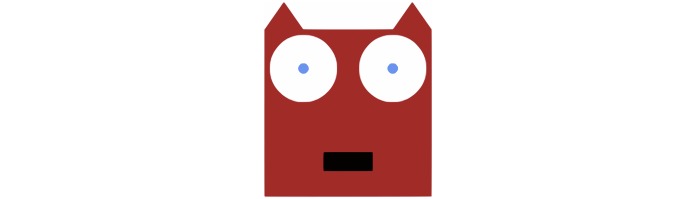
Example stimuli. Example of a demon used in the game.

The experiment was created using Flash web programming IDE; the program ran on client machines and intermittently sent data back to the lab webserver using the JSON protocol (source code is available on request). Demons differed on three dimensions: eye diameter, color of body, and horn height (see Fig 2 for an example). Eye diameter was a random number from a uniform distribution in the range (5 pixels, 35 pixels). Demon bodies were randomly assigned a color from the set {red, green}. For Conditions 2, 3, and 4, horn heights were 10 pixels for demons that went right and 50 pixels for demons that went left. For Condition 1, horn height was chosen from the same set, but it did not predict anything about the demon's behavior. In all cases, horn width was a constant 20 pixels.

### Procedure

Subjects began by reading instructions. All subjects were given a multiple choice quiz on each element of the instructions that they had read. Subjects who made any mistakes on this quiz were required to reread the instructions and retake the quiz, a process that was repeated until they responded perfectly. For subjects in Condition 1 and 2 (Control and Predictable/Uninstructed groups) the instructions described the goal of the game but said nothing about the location of reappearance of the demon. For subjects in Condition 3 (Dimension Instruction), the instructions stated “One important thing you should know: the height of the demon's horns predicts something about the demon's behavior.” For subjects in Condition 4 (Full Instructions), the instructions stated “One important thing you should know: the height of the demon's horns predicts whether the demon will re-appear on the left or right. Long-horned demons reappear on the left, and short-horned demons reappear on the right.” (Each of these elements was included on the comprehension quiz given to this group of subjects.)

Subjects then played for 180 trials (unless they achieved a perfect score on any block, at which point their play was terminated). Finally, all subjects (except those in the Full Instruction condition) saw a screen asking them to list up to three hunches they might have about how they could predict which direction the demon would go. For each response, the subject was asked to indicate a level of confidence (using increments of 10%.)

## Results and discussion

To be included in the analysis, subjects had to finish the experiment and answer the survey questions. Data from two subjects (one from the Full Instruction Condition, one from the Dimension Instruction Condition) who completed the experiment were dropped from further analysis because it turned out that they were missing data from more than four trials in one of the blocks due to internet connectivity problems. A few other subjects had missing trials for the same reason, but were missing fewer than four trials total. Those data were included in the analysis. There was a significant main effect of group, *F*(4, 97) = 21.76, *p* < 0.001, and block, *F*(5, 485) = 38.35, *p* < 0.001, as well as a significant interaction, *F*(20, 485) = 3.10, *p* < 0.001.

### Control group performance

**Fig 3:**
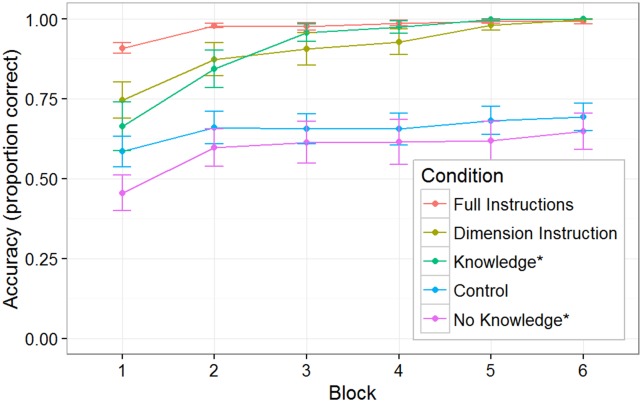
Experiment 1 subject performance. Proportion of plays resulting in successful scores for Control, Dimension Instruction, and Full Instruction Groups, as well as for two sub-groups of the Predictive/No-Instruction groups (Knowledge* subjects who were able to verbally report rule, and No-Knowledge* subjects who could not do so.) As described in text, for computing these averages, whenever subjects scored on 100% on a 30-trial block, they were assigned 100% score for computing average performance in the subsequent blocks. No-Knowledge* subjects scores are not superior to those of control subjects for whom there was no predictive relationship available to be exploited.

The Control group, for whom prediction was not possible, contained 21 subjects. In Fig 3, the line labeled Control Group shows the average performance of this group of subjects (as well as the standard error). The average performance shows no more than a very gradual rise in score level. Thus was expected, because when a subject has failed to anticipate the position of the demon (as would have been the case in at least 50% of plays), it is only occasionally possible to get the mouse over to the appropriate side of the screen rapidly enough to click on it before it disappears—and continued practice produces for most subjects only a limited improvement in score levels achieved by this strategy. A small fraction of subjects do manage this, however, and indeed, two of the 21 subjects (10%) in this condition reached the criterion of perfect performance on a block (neither reported any hunches about predicting the demon's trajectory). The Supplementary Online Materials (S1 File) show the verbatim exit-interview responses of the 17 subjects in this group who were willing to comment in response to the request to specify any rule they thought might possibly have predicted the direction the demon would go in (of course, the direction was actually chosen randomly.) The average confidence reported by the 17 subjects who provided hunches was 59%. Interestingly, there were many highly confident reports of predictive rules that had no valid correspondence to the rules that generated the stimuli (e.g., 70% confidence in “The bigger ears came from the right, whereas the smaller ears ones came from the left.” and 70% confidence in “the more i miss on one side is the more it goes to that side” and 90% confidence in “coming on to the end of each trial the demons would go either to the left or right about 3 to 5 times straight”.)

### Full instructions/dimension instructions group performance

As expected, subjects who were explicitly told the predictive rule or relevant dimension had near perfect mastery by the end of the game. Recall that this game is not a 2-alternative forced choice task, thus some variability can be introduced through motor errors or trials where responses were withheld by the subject.

### Predictable/No instructions group performance

The group whose performance is of greatest interest for examining behavior/awareness dissociations is the Predictable/No Instruction group, containing 26 subjects. Out of these, 14 (54%) were able to perform perfectly on a block of trials, allowing them to terminate their participation. The Supplementary Online Materials show the verbatim exit interview responses of all subjects in the Predictable/No-Instruction group, classified (blind to other aspects of the subject's behavior) according to whether the response indicated complete and accurate knowledge of the rule or not. Two subjects' hunch texts were judged unclassifiable because they contained partial bits of correct information combined with elements of misinformation, thus were excluded from analysis.

In Fig 3, the line labeled No-Knowledge* shows the performance of the 11 subjects judged to have shown no conscious knowledge of the rule. As can be seen, there is no indication that they perform any better than subjects in the Control Group for whom there was no predictive rule, *F*(1, 33) = 0.73, p = 0.40. If they learned anything about the predictive relationship present in the game, they evidently made essentially no use of that learning. Of these 11, one achieved perfect performance in a block, a rate (9%) very similar to the proportion of control subjects achieving this level.

In the Predictable/No-Instructions group, 13 subjects did succeed in describing the rule. All 13 of these Knowledge* subjects (100%) attained perfect performance in one of the blocks of trials. Their performance is shown with the line labeled Knowledge* in Fig 3. Note that for purposes of this graph, whenever subjects scored on 100% of a 30-trial block, they were assigned 100% score for all subsequent blocks—otherwise the rightmost points on graph would reflect an increasingly truncated sample as subjects are peeled off due to having reached perfect performance in an earlier block. (This decision seemed sensible because pilot experimentation in which subjects were required to complete all 6 blocks regardless of performance showed that after people once attained mastery in a block, they scored on close to 100% of trials thereafter; we suspect that the few failures reflected inattention caused by the boredom of performing a task that now lacked any challenge.)

### Individual knowledge* subjects’ performance

**Fig 4:**
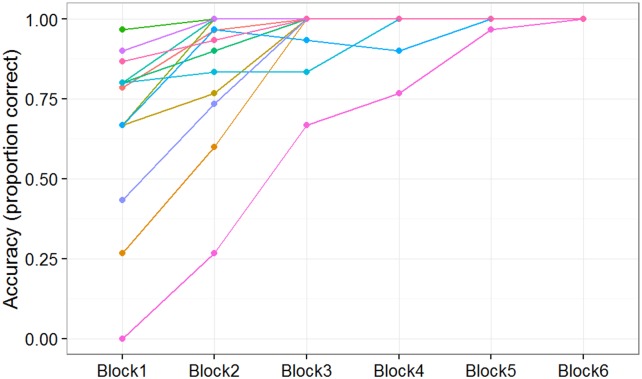
Individual subject performance for knowledge* group. Proportion of plays on which the subject scored as a function of block for subjects in the Knowledge* subset of the Predictable/No-Instruction group (subjects able to verbally report the predictive rule.) Each line shows a different subject from the group. All subjects in this group reached 100% performance in one of the blocks.

To provide a more fine-grained picture of the Knowledge* subjects' performance, Fig 4 displays the scores of all 13 individuals in this group block by block. Subjects are highly variable in their Block 1 performance. The figure also shows what appears to be relatively steady progress over the course of the session by most of the subjects who ultimately attain perfect mastery. The latter two statements are jointly confirmed by the strong negative correlation seen between (a) scores on Block 1 and (b) the block number in which the subject first reaches perfect performance (*r* = -0.63, *p* = 0.02).

### Trial of last error

**Fig 5:**
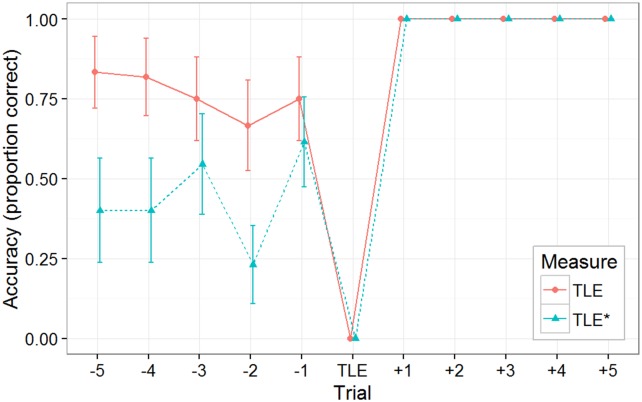
Experiment 1 TLE and TLE* performance. Performance for subjects in the Knowledge* subset of the Predictable/No-Instruction group for 24 trials prior to the subject's Trial of Last Error (TLE), averaging over pairs of adjacent plays. TLE(-5:-1) mean: 76%; TLE*(-5:-1) mean: 44%.

To provide a more fine-grained look at what precedes the “perfect mastery”, we examined subjects’ performance on 5 trials just prior to each individual’s trial of last error (TLE and TLE*; see [15], for an early study focusing on this measure in the context of concept learning). The TLE is specifically constrained to an individual’s last trial such that an incorrect response was made. Hence, averaged across all subjects, the TLE has a mean accuracy of 0 with no variability. Given that the TLE is defined as each individual’s last error in the experiment, all remaining trials must have perfect accuracy. The first TLE line (solid) shows performance hovering around 75%, followed (as must happen, given the definition of TLE) by a trial with zero accuracy (the last error) and then a performance of 100% on the remaining trials of the experiment. What is striking in the TLE is the relatively flat performance curve over this immediate pre-mastery period (see Fig 5), and the fact that the level of performance here (76%) is considerably higher than that seen overall in either the Control or the No-Knowledge* groups. (Superficially, the flatness of this figure might appear to paint a rather different picture of the buildup to insight than what is seen in Fig 3—but there is no conflict, because Fig 3 plots improvement over a far longer time-scale.) The TLE would suggest learners who ultimately attain mastery tend to reach that point by jumping up from a rather high plateau that already supports performance that is much better than what subjects in the Control and No-Knowledge* conditions generally ever attain.

However, after examining subjects’ cursor locations during the course of Experiment 2, we came to think that our early termination criteria (a completed block of 100%) might make that TLE potentially a bit misleading. Suppose a subject gained insight to the predictive feature relatively early in a block. They could still make a motor error (e.g., not moving or clicking the mouse fast enough to score the point), and indeed they would have rather little incentive to score perfectly on the remaining trials within that block (since the reward of leaving early was available only for a perfect score on an entire block). We suspect this is likely the case given the non-perfect scoring in the Full Instructions group.

When subjects made a motor error after attaining insight, the apparent TLE would be shifted relative to the “true TLE”. To deal with this subtle measurement pitfall, we computed another measure of the trial of last error, which we call TLE* (dashed). This is the last error that precedes five successive errorless trials (0.031 probability of occurrence by chance). The results of TLE* as rather convincing, in our opinion, in showing that the final mastery was accomplished *de novo* by jumping from a state of complete ignorance (44%) of where the demon will next appear.

## Experiment 2

To further investigate the surprising result where unawareness of the predictable feature led to performance that was no better than chance and final mastery was potentially de novo, we ran a higher powered version of the first experiment, this time specifically comparing Condition 1 (control) and Condition 2 (Predictive/No Instruction). In addition, we changed a few parameters to increase the difficulty of the game.

## Method

### Participants

Two hundred undergraduates from the same subject pool participated in this experiment for course credit. All were naïve to the purpose of the experiment.

### Stimuli

Stimuli were created using the same process as Experiment 1 with the following exceptions: Eye diameter determined direction of the demon {15px: Left, 30px: Right}. For the control condition, the eye diameter was randomly chosen from the discrete set {15px, 30px}. Eye color was randomly selected from the color set {yellow, blue} and assigned to 1 of 5 shades of the selected color. Horn height and width were drawn independently from a uniform distribution in the range [15 pixels, 60 pixels]. And lastly, the demon bodies were randomly assigned a color from the set {red, grey}.

### Procedure

The procedure was identical to Experiment 1 with the exception that Experiment 2 only included Condition 1 (Control) and Condition 2 (Predictive/No Instruction). In addition, the timing of a single trial was modified as follows: The V-shaped occluder appeared on screen alone for 2 seconds. Then the demon appeared at the bottom on the screen and paused for 3 seconds. After which, the demon would disappear behind the occluder for 2 seconds and reappear either or the left or right side of the occluder moving off screen with the demon being visible and clickable for 0.5 second. The trial concluded with a 2-second blank screen ISI.

## Results and discussion

A research assistant scored the hunches while being blind to the conditions. The first scoring criterion examined was that the subjects had to explicitly mention the predictive feature (i.e., the eyes). We also examined a stricter criterion where the statement had to include the idea of “big eyes to the right and small eyes to the left”. No reliable differences between the two scoring criteria were observed, so we used the first scoring criterion for the data described here. Of the 100 subjects in the predictive/no-instruction condition, 40 subjects were counted as aware (Knowledge*) while 60 subjects were scored as No-Knowledge* (25 reported an incorrect hunch and 35 reported no hunch).

### Block performance

**Fig 6:**
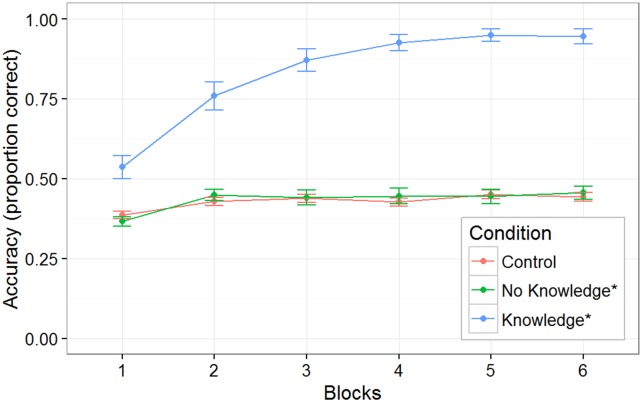
Experiment 2 subject performance. Proportion of plays resulting in successful scores for Control and Predictive/No-Instruction groups (Knowledge* subjects who were able to verbally report rule, and No-Knowledge* subjects who could not do so.) Averages were computed in the same manner as with Experiment 1. No-Knowledge* subjects scores were not superior to those of control subjects for whom there was no predictive relationship available to be exploited.

**Fig 7:**
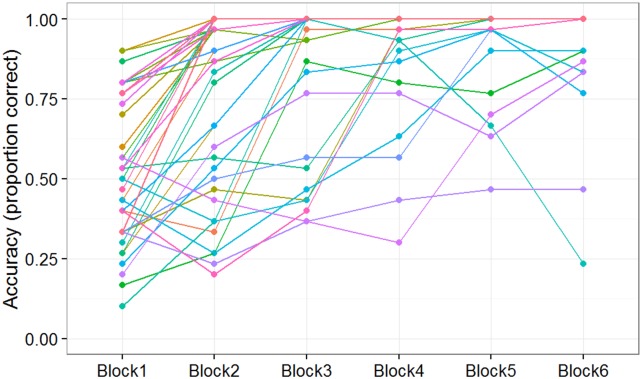
Individual subject performance for knowledge* group. Proportion of plays on which the subject scored as a function of block for subjects in the Knowledge* subset of the Predictable/No-Instruction group (subjects able to verbally report the predictive rule.) Each line shows a different subject from the group. The majority of subjects in this group reached 100% performance in one of the blocks.

Fig 6 shows the same pattern of results. There was a significant main effect of group, *F*(2, 197) = 186.7, *p* < 0.001, and block, *F*(5, 985) = 42.64, *p* < 0.001, as well as a significant interaction, *F*(10, 985) = 23.61, *p* < 0.001. In line with Experiment 1, subjects who successfully described the predictive rule were able to reach a high level of performance while subjects that were unable to verbalize the predictive rule did not perform better than the control subjects, *F*(1, 158) = 0.07, *p* = 0.79. Subjects in the Knowledge* group did have more variability in performance (see Fig 7) in which some subjects were unable to complete at least one block at 100%. We would attribute this to the higher difficulty level of the game (i.e., faster demon exit speed).

### Trial of last error

**Fig 8:**
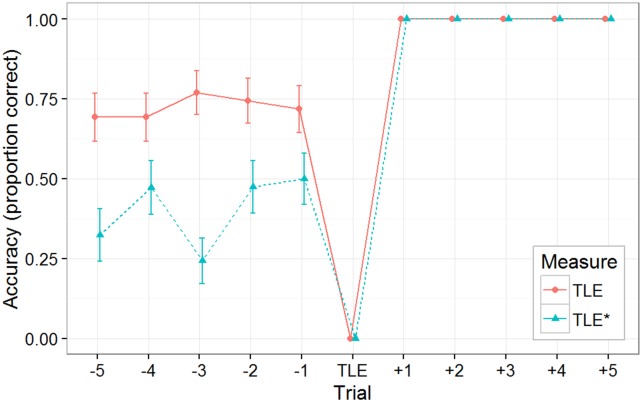
Experiment 2 TLE and TLE* performance. Performance for subjects in the Knowledge* subset of the Predictable/No-Instruction group for 12 trials prior to the subject's TLE, averaging over pairs of adjacent play. TLE(-5:-1) mean: 72%; TLE*(-5:-1) mean: 40%.

The results from the TLE and TLE* analyses (see Fig 8) as described in Experiment 1 Results were strikingly similar to what was seen in Experiment 1. Performance prior to the TLE (solid) was relatively high (72%) given the stringent early termination criteria. However, as discussed above, the results of TLE* (dashed) indicate that the final mastery was again accomplished *de novo* by jumping from a state of complete ignorance (40%) of where the demon will next appear.

### Cursor placement

**Fig 9:**
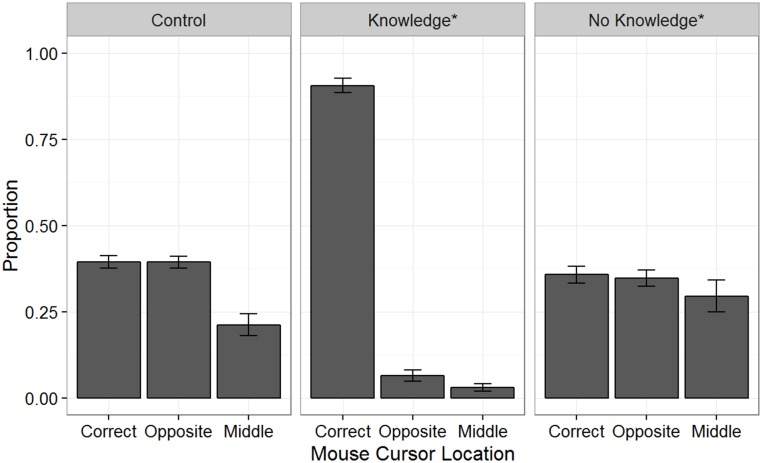
Cursor locations. Proportion of cursor locations just prior to the demon exit for the last two blocks of the game. The screen was divided into thirds: Correct (i.e., mouse cursor was on the side that the demon will exit), Opposite (i.e., the mouse cursor was on the opposite side that the demon will exit), and Middle (i.e., the mouse cursor was in the middle 1/3 region).

To uncover the relationship between awareness and exploitation of the predictive feature in more fine grain, we also looked at where subjects placed the cursor just prior to the demon’s exit of the occluder in each subject’s last two blocks of plays. This gives us a more sensitive measure of subjects’ predictions prior to making a committed response (i.e., the mouse click). Three possible ways subjects might play this game are to: (1) intentionally choose an exit based on a hunch, (2) randomly choose an exit, or (3) place the cursor in the middle to minimize the distance between the exits and the cursor. If subjects learned the predictive feature, either explicitly or implicitly, one would expect that they are more likely to use this to their advantage by placing the cursor at the correct tunnel exit before the demon exits the tunnel. Given the two distinctive strategies (side- and middle- choosing), the game screen was sectioned into equal thirds (Correct Side, Opposite Side, Middle) by pixels along the x-axis. For example, if a demon came out of the left tunnel, a mouse cursor on the left third of the screen would be labeled “Correct”, a mouse cursor on the right third would be labeled “Opposite”, and a mouse cursor in the middle third would be labeled “Middle”. Fig 9 shows the proportion of cursor location by Group and cursor location for the last two blocks of game play. Across the 3 groups, cursor location was statistically significant, *F*(2, 388) = 58.66, *p* < 0.001.

For the Control group, there was a significant difference in where the mouse cursor was located, *F*(2, 198) = 13.98, *p* < 0.001. However, a post-hoc bonferroni-corrected pairwise t-test revealed subjects did not exhibit any correctly anticipate the side where the demon would appear (Correct vs. Opposite, *p* = 1). This was of no surprise and was to be expected given no predictive feature to be exploited.

For the Knowledge* group, there was a significant difference in where the mouse cursor was located, *F*(2, 78) = 596.2, *p* < 0.001. A post-hoc bonferroni-corrected pairwise t-test revealed significant differences for Correct vs. Opposite (*p* < 0.001) and Correct vs. Middle (*p* < 0.001) and no significant difference for Opposite vs. Middle (*p* = 0.21). In other words, Knowledge* subjects were able to exploit the predictive feature (i.e., small eyes and big eyes) by placing the cursor in the correct location (i.e., left and right, respectively) in anticipation of the exiting demon. In addition, we examined the cursor locations for the trials preceding TLE*. We would predict that if conscious insight did occur at the TLE*, the cursor locations for the preceding TLE* trials would not differ. This post-hoc analysis revealed no difference in cursor locations, *F*(2, 76) = 1.86, *p* = 0.16, suggesting there was no insight prior to the TLE*.

Lastly, subjects in the No-Knowledge* group did not seem to anticipate where the demons would exit, *F*(2, 112) = 0.67, *p* = 0.51. While these data (e.g., Correct vs. Middle, *p* = 1; Opposite vs. Middle, *p* = 1) show a different pattern compared to the Control group (e.g., Correct vs. Middle, *p* = 0.001; Opposite vs. Middle, *p* = 0.001), we suspect individual subjects used a combination of the side-guessing and neutral-region strategies to different degrees.

## General discussion

The studies reported here examined what happens when subjects play a simple video game that embodies a discrete and very useful predictive relationship (the height of a demon's horns, Exp. 1, or diameter of the demon’s eyes, Exp. 2, predicts which way the demon will “choose” to go, information the player can exploit by laying in wait for the demon). Of most interest was what happened for a group of subjects who were not given any hints about the existence of the predictive regularity that they were exposed to (the Predictable/No-Instruction Condition). Based on the final exit-interview reports, this group of subjects could be divided surprisingly cleanly into two different subgroups: those who demonstrated clear (and verbalizable) conscious access to the rule (the Knowledge* subgroup), and those who did not (No-Knowledge* subgroup), with only a few subjects resisting easy classification.

Whereas past studies of implicit learning have generally found only a very weak or nonexistent relationship between conscious insight and behavioral indices of implicit learning, here the scores attained by the two groups very closely tracked their ability to verbally articulate the hidden rule. Indeed, for those showing no conscious access, there was no evidence that they acquired any ability whatsoever to make use of the predictive relationship in their game play. Their scores remained comparatively to the performance level of control subjects playing a random version of the game that did not allow prediction of where the demons would reappear. By contrast, the Knowledge* group, who were able to articulate the rule, showed dramatically better overall scores, almost all reaching the criterion of perfect performance within a whole block. In addition, Knowledge* subjects appear to accomplish this de novo and not from gradual learning (i.e., an abrupt jump in performance as well as a change in cursor placement behavior in pre-TLE* and post-TLE* trials).

While one might argue that accuracy is not sensitive enough show implicit learning and measurements such as reaction time must be used, our examination of the cursor data in Experiment 2 seem to shed some light on the No-Knowledge* subjects. Had there been any implicit learning, we would expect there to be at least a bias in where cursors were placed prior to any overt decision response of a mouse-click. Yet the pattern of nearly all No-Knowledge* subjects’ cursor locations were in all three regions regardless of a demon’s exit direction.

### Limitations and connections to previous findings

As noted above, the tight linkage between awareness and implicit usage of the regularity in the present study appears to conflict with the conclusion of several previous lines of research on implicit learning and awareness (e.g., [4,10,16]). Why is this? One possible reason is that the learning revealed in the earlier studies is encapsulated within the motor or perceptual systems. By contrast, in the game used in the current studies, useful learning based on the regularity would seem to require learning to base an action on the crucial predictor feature, a strategy change not just a modulation of response latency. Evidence for such unconscious learning is strikingly absent. One possibility, as noted earlier, is that the fact that the regularities embedded in Willingham et al. [4] and Miller [10] studies were only partially valid suppressed conscious utilization of these effects. Each of the differences noted suggests potentially testable hypotheses for follow-up research.

While the results challenge any assumption that implicit learning in general is unconscious, powerful, and “cognitively impenetrable”, it is important not to overstate the conclusions. It is possible that with more training, non-conscious learning might have developed in this situation to take advantage of the predictive regularity embedded in our game. Perhaps this had even begun operating far too slowly to have produced any results that could be detected within the time limits of this study. Thus, it is possible that, like the proverbial tortoise, such a putative non-conscious learning process might eventually have caught up and enabled the player to exploit the predictive relationship without any conscious awareness of the relationship. This, too, is a testable interpretation.

A second limitation is that the results do not clearly indicate whether the learning process revealed here depends upon active and conscious hypothesis-testing, e.g., consciously and explicitly checking the relationship between each feature of the demon and the demon's behavior. It could be that the link to awareness exists because the learning arises from such a conscious reasoning process. Alternatively, as noted above, it could be that a slow buildup of information in a non-conscious learning system results in conscious awareness once a sufficiently high level of predictive success has been achieved. This kind of interpretation was suggested some years ago by Boakes in discussing the tight linkage between awareness and Pavlovian conditioning (see [17,18]). As mentioned above, the detailed time-course of learning here arguably fits somewhat better with the latter interpretation than it does with the conscious hypothesis-testing interpretation.

One intriguing aspect of the present results is the fact that subjects in the control condition often reported apparently illusory rules for the behavior of the demon (see Supplementary Online Materials) and in some cases, they voiced these with strong confidence. Future research might shed light on the interplay of conscious and non-conscious learning processes by examining in detail the stimulus displays seen by subjects who claimed to have discovered rules that are (at least on expectation) false. One question of interest is whether these reports reflect unusual local statistics of what these particular subjects actually experienced, or instead are completely fanciful.

A third limitation of the current study is that (quite by design) it used a predictive relationship that is potentially easy to verbalize. Naturally, predictive relationships that are not so easily verbalized (e.g., acquiring dexterity with motor skills like tennis playing and driving for which most people probably have only a poor descriptive vocabulary) may not show such a close connection to awareness (cf. [19]).

These limitations notwithstanding, the results of the present work lead to several conclusions. One is that it is possible to embed predictive regularities in computer games and to track their behavioral exploitation (comparing it to conscious reports), thus offering new ways to examine implicit learning using behavioral measures more compelling than modulation of reaction time. Second, given a highly reliable and useful predictive regularity in a game, the behavioral exploitation of this regularity can sometimes emerge with a far stronger linkage to conscious awareness than has generally been noted in the implicit learning field.

## Supporting information

S1 File Subjects’ exit responses. (DOCX)
