# Supplementary material for: Learning to exploit a hidden predictor in skill acquisition: Tight linkage to conscious awareness
Source: PLoS One. 2017 Jun 20;12(6):e0179386. doi: 10.1371/journal.pone.0179386 (PMC5478109; doi:10.1371/journal.pone.0179386)
Supplement: S1 File — (DOCX) [file pone.0179386.s001.docx]

Randy Tran* and Hal Pashler

*To whom correspondence should be addressed. Email: r4tran @ ucsd .edu

**Experiment 1**

“Hunches” about rule for demon travel by subjects in the Control Condition (where the demon's motion was completely random). List contains verbatim responses of all subjects who responded.

1. based on the horns and the eyes size
2. At first I thought it was a color linked system where if the same color came twice it would shift. This was wrong.
3. The smaller eyes ones tend to come from the left and the bigger eyes ones came from the right.
4. if i get two right on one side it goes to the othere side
5. first I guess blue to right red to left
6. no particular pattern, very randomly
7. i thought reds go right blues go left.
8. I thought there was a correlation betwen the demon's mouths and which way they were going.
9. blue to the left red to the right
10. did not see any
11. coming on to the end of each trial the demons would go either to the left or right about 3 to 5 times straight
12. Each color had 6 types (2 eye types and 3 horn types). Each type would alternate which side they would exist.
13. I noticed at first that some demons were; blue, red, little horns, big horns, little eyes, big eyes, smile, not smiling.
14. None.
15. at first i tried to count but it didnt work. i think it is more luck then anything
16. I did not notice any rule regarding which kinds of demons tended to go in which direction.
17. Red usually go to the right and Blue usually go to the left.
18. [No hunch provided]
19. [No hunch provided]
20. [No hunch provided]
21. [No hunch provided]

“Hunches” about rule for demon travel provided by subjects in the Predictable/No-Instruction Condition (for these subjects, long-horned demons always went left and short-horned demons always went right, although the subject was not informed of this rule.) List provides verbatim responses of all subjects.

Responses Judged Incorrect

1. Reds seemed to alternate more; Blues seemed to go the same way more often
2. Sometimes, it seemed like the demons went to the left for three times and then to the right for three times.
3. no clue
4. No hunches really. Thought about it briefly, but didn't postulate anything
5. No I didnt but there was a pattern
6. i did not really pay attention to the rules of which way they go
7. the demon with long horns always go to my right
8. blues to right and reds to right; first sided always the maximum
9. I thouoght that the red demons were moving to the left, but that didn't hold true throughout the experiment.
10. [No hunch provided]
11. [No hunch provided]

Responses Judged Correct

1. Long horns go to the left; Short horns go to the right
2. demons with short one goes to the right and the long one goes to the left
3. Tall eared 'demons' went left facing me on screen while short eared 'demons' went right.
4. Demons with larger horns went left; those with smaller horns went right.
5. Bigger ears to one side, smaller to the other
6. demons with big horns went left and demons with small horns went right
7. The demons with long horn went left, with short horn right.
8. the ones with long horns tend to go to the left and the ones with the short horns tend to go tothe right.
9. tall ears go left--short ears go right
10. yes taller horns left
11. Demons with long horns went to the left side of the tunnel, and demons with short horns went to the right side.
12. demons with tall horns go left, those with short horns go right
13. Demons with bigger horns go to the left.

Responses Judged Unclassifiable

1. The blue demons tend to go left; The red demons with small horns tend to go right; The red demons with big horns tend go to left.
2. the blue, short-horned demon went right.

**Experiment 2**

“Hunches” about rule for demon travel provided by subjects in the Predictable/No-Instruction Condition (for these subjects, small-eyed demons always went left and big-eyed demons always went right, although the subject was not informed of this rule.) List provides verbatim responses of all subjects.

Responses Judged Incorrect

1. I think they tend to go to the right firdt, then after a few times going to the right I think they go to the left.
2. If a lot of the demons have gone to the right, I assume the next one will go to the left.
3. They usually go to the right
4. After 3 demons goes to the same way, there is a high possibilitiy that the next one will go the other way
5. The color of the eye and the color of the demon\'s face kinda have a pattern. But it\'s not always right
6. I thought that the specific characteristics of the demons such as eye color, horn size, body color, and mouth shape may have had a role in determining
7. I started basing my hunches on eye color and demon color. Generally, if it\'s blue eyes and red, it goes left and if it\'s yellow eyes and gray, it go
8. The red demons go right, while the gray demons go left. It does not happen all the time, but it sometimes does.
9. Bright blue, red, cut
10. I assume the demon is going to come out from the right side.
11. when a demon of particular characterisitcs goes into the tunnel and then comes out of one of the upper sides, lets say upper left, the next time it ap
12. My hunch is based on the eyes colors and the demon\'s body color.
13. Maybe it has something to do with eye color
14. eye color and body color
15. Red moves right mostly, gray moves left mostly
16. demon\'s mouth shape determine which direction it goes.
17. Demons switch directions every for 4-5 demons
18. color of the demon
19. Originally I thought all yellow eyed demons went left and all blue eyed demons went right. That worked for a bit but then stopped.
20. the demon may go the same way many times
21. Red ones will go right
22. Narrower and smaller ears tend to go to the right
23. The red one with large white eyeballs with blue small pupils will go to the right.
24. Those with blue eye color will most likely move to the right
25. usually move left first then switch back and forth, ends on left
26. [No hunch provided]
27. [No hunch provided]
28. [No hunch provided]
29. [No hunch provided]
30. [No hunch provided]
31. [No hunch provided]
32. [No hunch provided]
33. [No hunch provided]
34. [No hunch provided]
35. [No hunch provided]
36. [No hunch provided]
37. [No hunch provided]
38. [No hunch provided]
39. [No hunch provided]
40. [No hunch provided]
41. [No hunch provided]
42. [No hunch provided]
43. [No hunch provided]
44. [No hunch provided]
45. [No hunch provided]
46. [No hunch provided]
47. [No hunch provided]
48. [No hunch provided]
49. [No hunch provided]
50. [No hunch provided]
51. [No hunch provided]
52. [No hunch provided]
53. [No hunch provided]
54. [No hunch provided]
55. [No hunch provided]
56. [No hunch provided]
57. [No hunch provided]
58. [No hunch provided]
59. [No hunch provided]
60. [No hunch provided]

Responses Judged Correct

1. big eyes= right, small eyes=left
2. Big eyes to the right and little eyes to the left.
3. The big eyed demon will go to the right and the small eyed demon will go to the left.
4. The demon with large eyes goes to right whereas the demon with small eyes goes to left.
5. Demons with the large eyes go to the right, while demons with the small eyes go to the left
6. The size of the demon\'s sclera determined which side it will reappear from. If the sclera was small, then the demon will move Left, if it was large,
7. Small eyed demons = appear on the left side / Big eyed demons = appear on the right side
8. Bigger eyes move to the left and smaller eyes move to the right
9. It\'s based on eye size. The ones with big eyes go right and the ones with little eyes go left.
10. size of eyes
11. The demons direction is determined by the size of their eyes. If they have small eyes, they will move towards the left and if they have big eyes, they
12. The demons with big eyes will move to the right, and the demons with small eyes will move to the left.
13. Demons with small eyes move to the left and demons with large eyes move to the right.
14. The demon moves right when its eyes are bigger and left when its eyes are smaller.
15. based on eye size
16. demons move right when they have large eyes and left when they have small eyes
17. The demons with large eyes got to the right and the demons with the smaller eyes go to the left.
18. The demons will move right if the eyes are big and will move left if the eyes are small
19. I looked at the size of the eyes and if they went right or left
20. Left is small eyes. Right is big eyes.
21. Big eye demons go to the right and small eye demons go to the left.
22. The big eye demons left from the right side and the small eyed demons from the left side.
23. the demons with the larger eyes go to the right, while the demons with the smaller eyes go to the left.
24. eye size
25. Large eyes go to the right; small eyes go to the left.
26. The demons with the small eyes go to the left and those with big eyes go the right.
27. The size of the demon\'s eyes indicates whether left or right. small eyes=left and large eyes=right
28. The direstion depends on the size of the eyes. Large eyes goes to the right and small eyes goes to the left.
29. If the demon has big irises, the demon will go right. If it has small irses, it will go left.
30. It also seems as though the demons with bigger eyes go to the right while the demons with smaller eyes go to the left.
31. I know that a demon will move to the right side if it has big eyes and it will move to the left side if it has smaller eyes.
32. Big Eyes go left. Small ones go right
33. There are two different eye sizes of the demons. If the demon has a relatively small eye size, it will leave out of the top left. If it has a relatively large eye size, it will leave out of the top right.
34. The eyes of the demon signals where the demon will appear. Smaller eyes = upper left, and larger eyes = upper right.
35. Demons with smaller eyes move to the left tunnel while the demons with larger eyes move to the right.
36. Big eyes = Right; Small Eyes = Left
37. The ones with the small eyes come out of the left side. The ones with the big eyes come out of the right side.
38. demons with large eyes move to the right, demons with small eyes move to the left
39. Small eyes to the left, big eyes to the right
40. Big eyes right. Small eyes left
